# Supplementary material for: BL-Hi-C is an efficient and sensitive approach for capturing structural and regulatory chromatin interactions
Source: Nat Commun. 2017 Nov 20;8:1622. doi: 10.1038/s41467-017-01754-3 (PMC5696378; doi:10.1038/s41467-017-01754-3)
Supplement: Supplementary file 1 — Supplementary Information [file 41467_2017_1754_MOESM1_ESM.pdf]

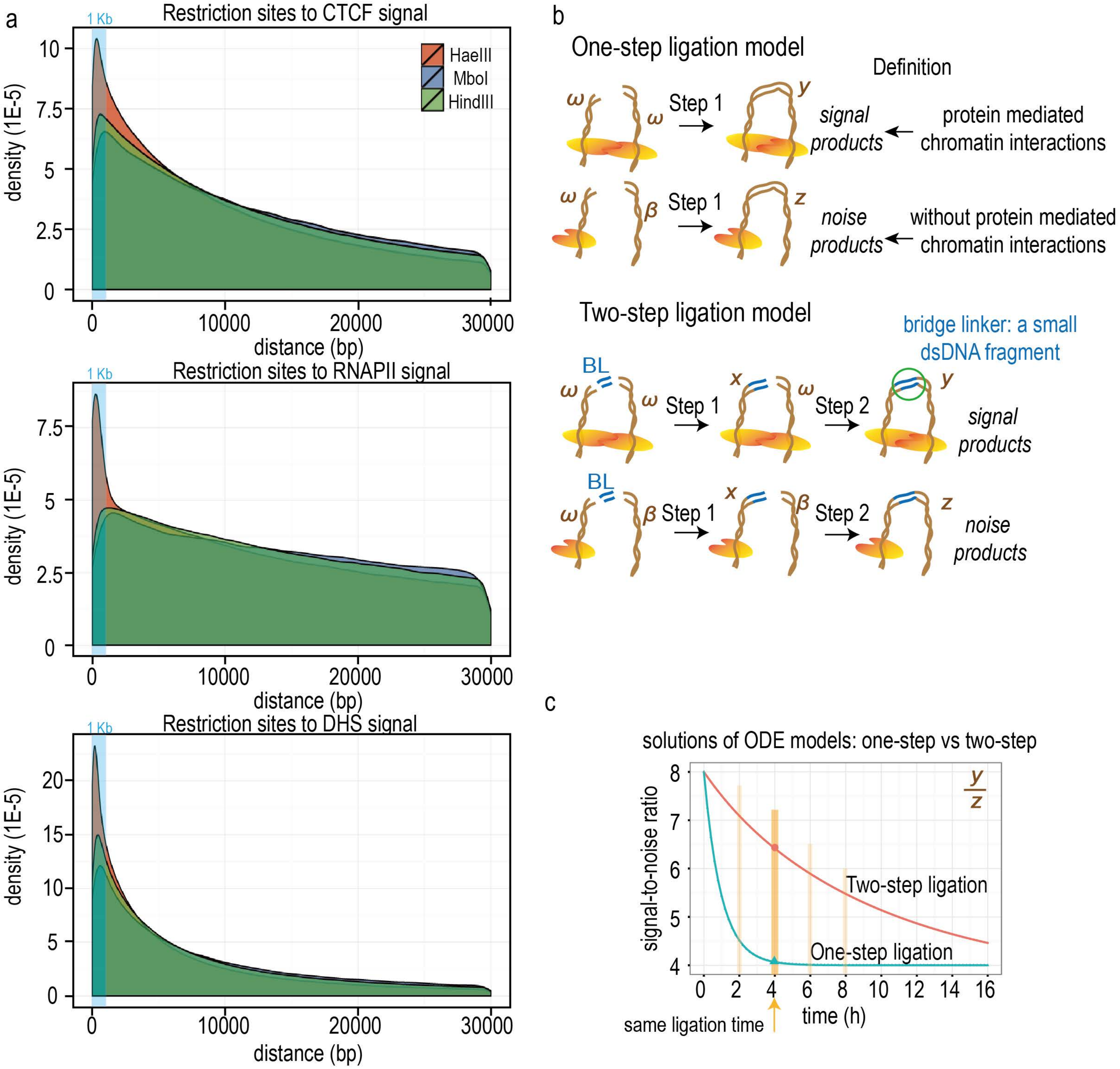

Supplementary Figure 1: Enrichment models for BL-Hi-C.

(**a**) Statistical distance between restriction sites (enzyme HaeIII, MboI and HindIII) and protein binding signals (CTCF and RNAPII binding signals and DNase I hypersensitivity sites). (**b**) Theoretical ligation models. In the one-step ligation model, double-stranded DNA (brown lines) interacts with binding proteins (orange), and the two fragments are ligated to form chromatin interactions defined as signal (mediated by proteins, orange) and noise (others) In the two-step ligation model, the fragment is joined with a bridge linker to form an intermediate product and then joins with another fragment, much as in the one-step reaction. (**c**) Simulation result for the ligation model. See the ordinary differential equation (ODE) in Methods. The X-axis refers to the ligation process and the Y-axis refers to the product ratio of signal and noise. The simulation result shows that for the same ligation time (4 hours), the two-step ligation method obtains a higher signal-to-noise ratio than the one-step ligation method.

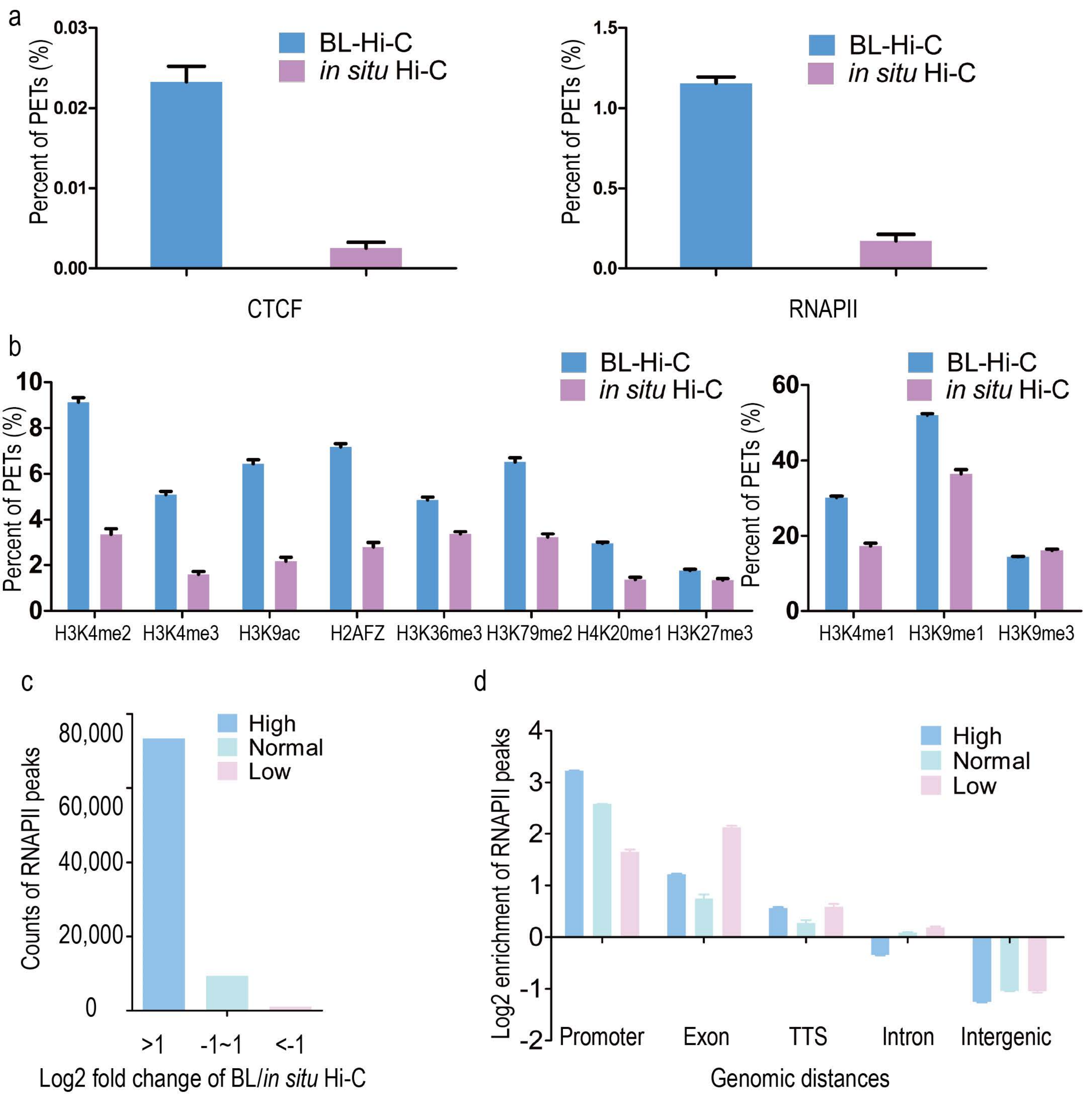

Supplementary Figure 2: PET enrichment of BL-Hi-C.

(**a**) The percentage of BL-Hi-C (blue) and *in situ* Hi-C (Rao *et al.* purple) PETs that are consistent with CTCF and RNAPII ChIA-PET loops on both anchors. (**b**) The percentage of PETs that are consistent with public ChIP-seq data on histone modifications. (**c**) The classification of public RNAPII peaks into high, normal and low, according to whether the fold change between BL-Hi-C and *in situ* Hi-C (Rao *et al.*) is > 1, -1 to 1 or < -1. The Y-axis refers to the counts of RNAPII peaks for each group. (**d**) The enrichment of high (blue), normal (green) and low (pink) groups of RNAPII peaks from BL-Hi-C at genomic features, according to the homer results.

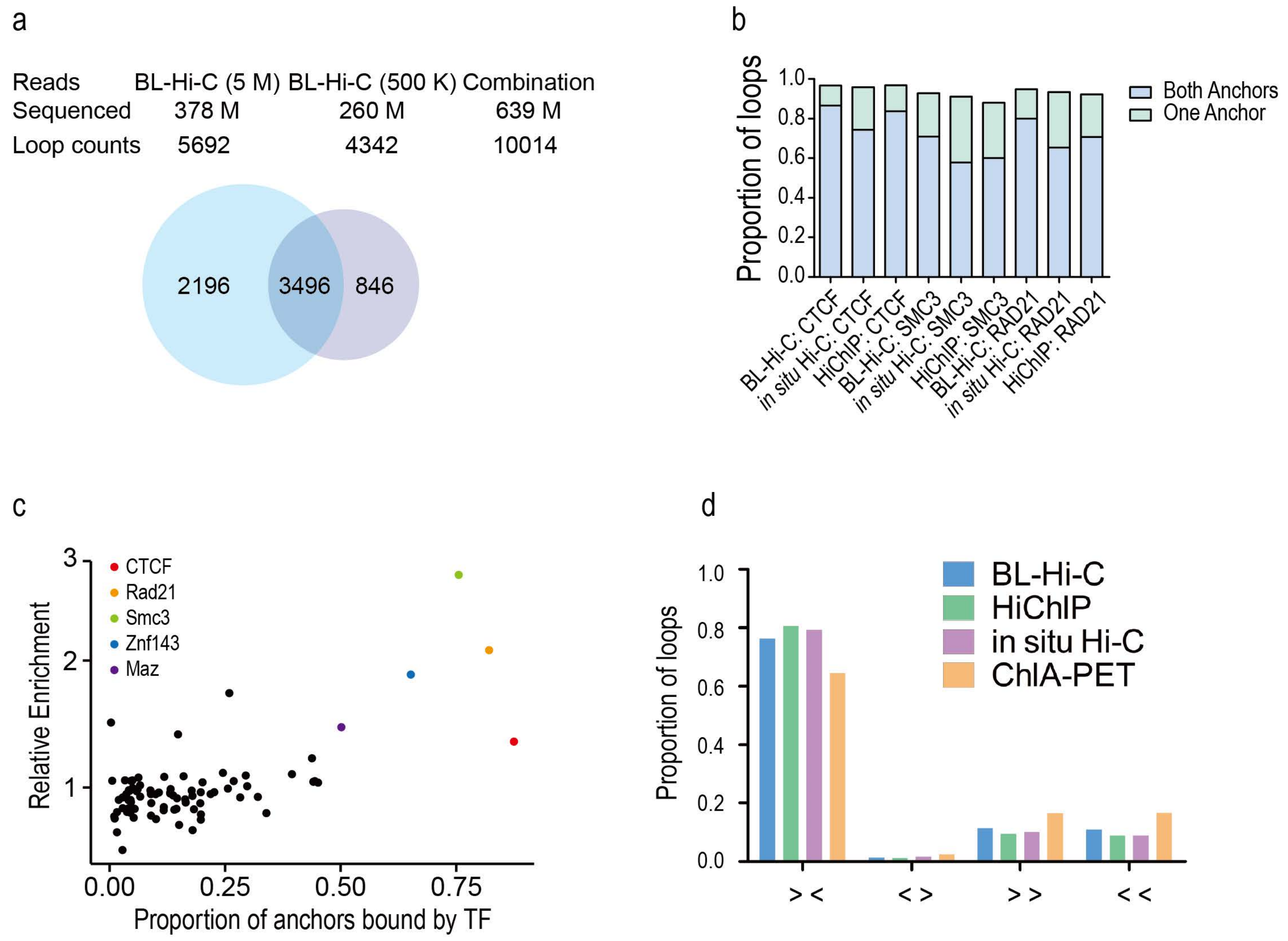

Supplementary Figure 3: Validation of BL-Hi-C loops.

(**a**) The chromatin loops identified in two BL-Hi-C datasets, 5 M and 0.5 M cells. (**b**) The proportion of loops that are consistent with the ChIP-seq data on CTCF, SMC3 and RAD21 obtained using *in situ* Hi-C (Rao *et al.*) and HiChIP. (**c**) The motif analysis at BL-Hi-C loop anchors. (**d**) The CTCF motif orientation of chromatin loops determined by BL-Hi-C, *in situ* Hi-C (Rao *et al.*), HiChIP and ChIA-PET.

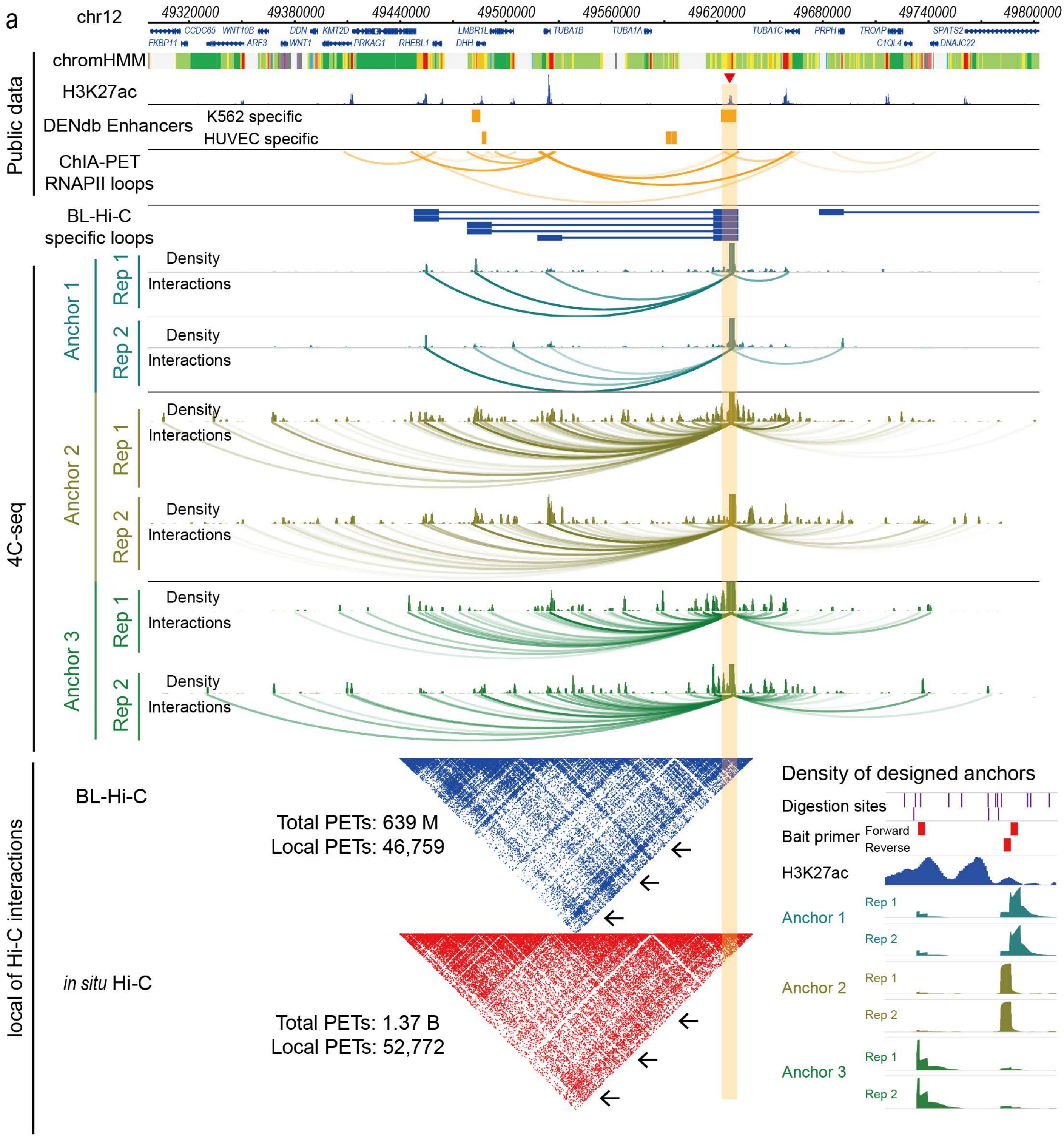

Supplementary Figure 4: Validation of BL-Hi-C-specific loops.

(**a**) The enhancer interactions are indicated by public data including ChromHMM, H3K27ac ChIP-seq, and DENdb-predicted cell type-specific enhancers and RNAPII ChIA-PET loops. The 4C-seq is applied to the enhancer anchor with three baits. The local interactions of BL-Hi-C (blue) and *in situ* Hi-C (Rao *et al.* red) are extracted. The data are visualized in the WashU Epigenome Browser.

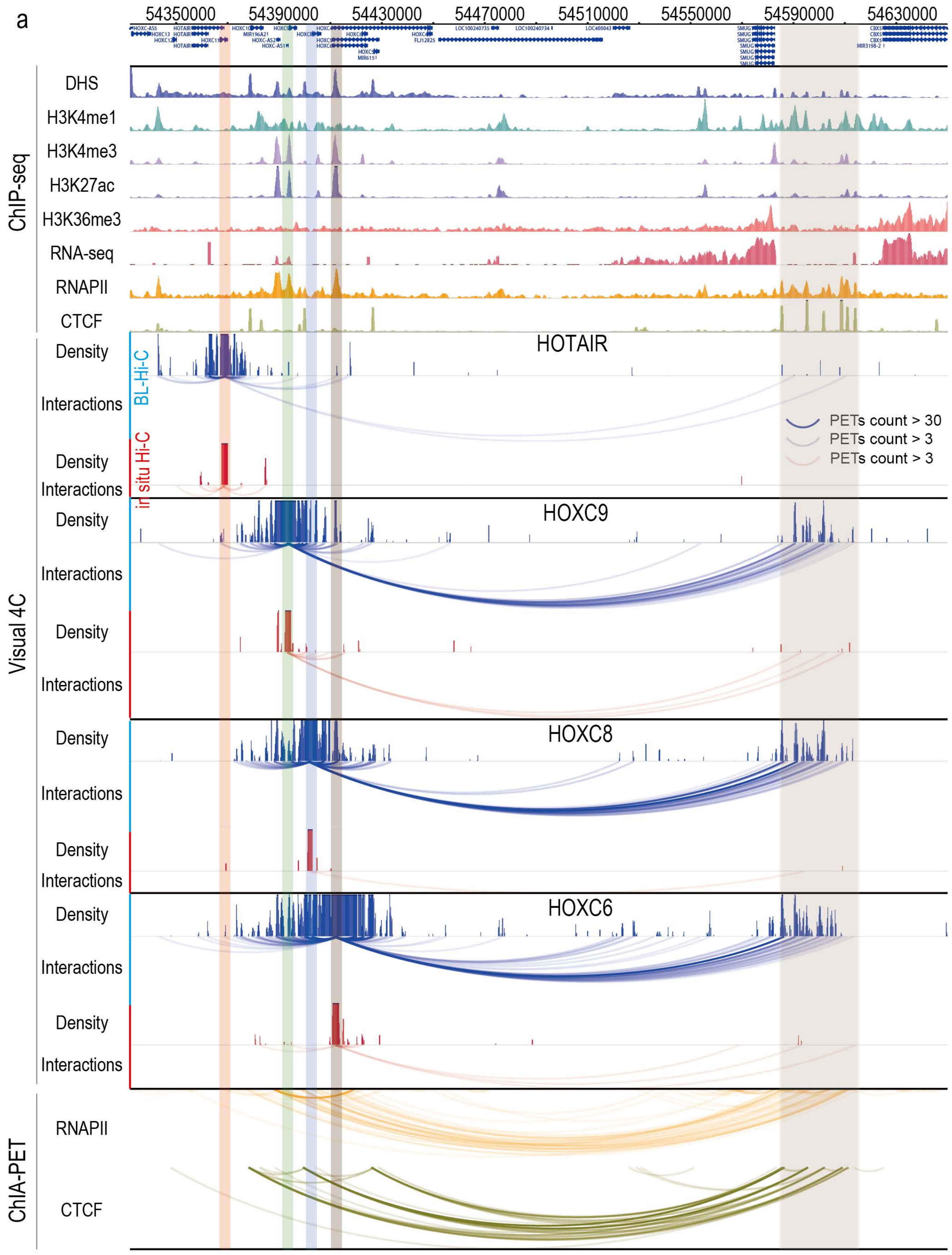

Supplementary Figure 5: Visual 4C on HOXC region.

(**a**) The narrow shadows indicate the viewpoints of HOTAIR, HOXC9, HOXC8 and HOXC6. The broad shadow indicates the known enhancer region. The arcs connect the viewpoints and interaction peaks in the Hi-C data. The public ChIA-PET data on CTCF and RNAPII represent the validated chromatin loops.

Supplementary Table 1:
The sequencing information of BL-Hi-C and public *in situ* Hi-C and HiChIP

Sample	Cell Type	Rep*	Protocol	Enzyme	Ligation	Cells	Total Reads
BL01	K562	1	BL-Hi-C	HaeIII	Two-step	5×10^6	8,124,484
BL01	K562	1	BL-Hi-C	HaeIII	Two-step	5×10^6	60,607,777
BL01	K562	1	BL-Hi-C	HaeIII	Two-step	5×10^6	98,688,232
BL01**							167,420,493
BL02	K562	1	BL-Hi-C	HaeIII	Two-step	5×10^5	7,306,054
BL02	K562	1	BL-Hi-C	HaeIII	Two-step	5×10^5	41,153,913
BL02	K562	1	BL-Hi-C	HaeIII	Two-step	5×10^5	109,108,537
BL02**							157,568,504
BL03	K562	2	BL-Hi-C	HaeIII	Two-step	5×10^6	103,581,192
BL04	K562	3	BL-Hi-C	HaeIII	Two-step	5×10^6	107,889,420
BL05	K562	2	BL-Hi-C	HaeIII	Two-step	5×10^5	103,410,494
BL06	K562	1	BL-Hi-C***	MboI	Two-step	5×10^5	24,051,814
BL07	K562	2	BL-Hi-C	MboI	Two-step	5×10^5	39,129,090
BL08	K562	1	BL-Hi-C***	HindIII	Two-step	5×10^5	31,739,876
BL09	K562	2	BL-Hi-C	HindIII	Two-step	5×10^5	58,094,739
BL10	K562	1	BL-Hi-C****	HaeIII	One-step	5×10^5	102,987,963
BL11	K562	2	BL-Hi-C	HaeIII	One-step	5×10^5	100,613,870
BL12	K562	1	BL-Hi-C	HaeIII	Two-step	5×10^5	101,396,869
BL13	K562	2	BL-Hi-C	HaeIII	Two-step	5×10^5	101,588,242
HIC069	K562	1	*in situ* Hi-C	MboI	One-step	5×10^6	456,757,799
HIC070	K562	2	*in situ* Hi-C	MboI	One-step	5×10^6	591,854,553
HIC071	K562	3	*in situ* Hi-C	MboI	One-step	5×10^6	79,905,895
HIC072	K562	4	*in situ* Hi-C	MboI	One-step	5×10^6	79,578,049
HIC073	K562	5	*in situ* Hi-C	MboI	One-step	5×10^6	77,353,816
HIC074	K562	6	*in situ* Hi-C	MboI	One-step	5×10^6	80,778,733
2138324	GM12878	1-1	HiChIP	MboI	One-step	2.5×10^7	160,399,050
2138325	GM12878	1-2	HiChIP	MboI	One-step	2.5×10^7	106,529,038
2138326	GM12878	2-1	HiChIP	MboI	One-step	2.5×10^7	212,384,008
2138327	GM12878	2-2	HiChIP	MboI	One-step	2.5×10^7	164,332,898
INL1	H9	1	*in situ* Hi-C	HindIII	One-step	5×10^7	34,860,295
INL2	H9	2	*in situ* Hi-C	HindIII	One-step	5×10^7	33,500,802

*The biological replicates (1-1 indicates biological-technical replicate).
**The combined dataset that sequenced in three times
***Modified BL-Hi-C protocol for alternative restriction enzymes.
****Modified BL-Hi-C protocol for alternative proximity ligation.

Supplementary Table 2: Public data that used

Experiment	Cell Type	Target	Accession ID
ChIA-PET	K562	RNA Pol II	GSM832464
ChIA-PET	K562	RNA Pol II	GSM832465
ChIA-PET	K562	CTCF	ENCSR000CAC
ChIP-seq	K562	H3K4me3	ENCFF752ALB
ChIP-seq	K562	H3K79me2	ENCFF350GQM
ChIP-seq	K562	H3K9me3	ENCFF323KXF
ChIP-seq	K562	H3K36me3	ENCFF784HLI
ChIP-seq	K562	H3K9ac	ENCFF418BIN
ChIP-seq	K562	H2AFZ	ENCFF681OSA
ChIP-seq	K562	H4K20me1	ENCFF363YMD
ChIP-seq	K562	H3K9me1	ENCFF001SZM
ChIP-seq	K562	H3K4me2	ENCFF627TIZ
ChIP-seq	K562	H3K4me1	ENCFF001SZH
ChIP-seq	K562	H3K27me3	ENCFF908KJV
ChIP-seq	K562	H3K9me3	ENCFF000BYU
ChIP-seq	K562	IgG-control	ENCFF000YQO
ChIP-seq	K562	IgG-control	ENCFF000YQP
ChIP-seq	K562	POLR2A	ENCFF000YWP
ChIP-seq	K562	POLR2A	ENCFF000YWR
ChIP-seq	K562	CTCF	ENCFF000YLT
ChIP-seq	K562	CTCF	ENCFF000YLU
ChIP-seq	K562	POL2A	ENCFF002CXQ
ChIP-seq	K562	CTCF	ENCFF002CWL
ChIP-seq	GM12878	POL2A	ENCFF002CPG
ChIP-seq	GM12878	CTCF	ENCFF002COQ
ChIP-seq	H1-hESC	POL2A	ENCFF002CJE
ChIP-seq	H1-hESC	CTCF	ENCFF002CIU
ChromHMM	K562	Chromatin state	HmmK562*
ChromHMM	GM12878	Chromatin state	HmmGm12878
ChromHMM	H1-hESC	Chromatin state	HmmH1hesc

*http://hgdownload.cse.ucsc.edu/goldenPath/hg19/encodeDCC/wgEncodeBroadHmm

Supplementary Methods

Reagents

Formaldehyde Solution (36.5~38% (wt/vol); Sigma-Aldrich, cat. no. F8775-500ML)

Glycine (Sigma-Aldrich, cat. no. G8898-500G)

Triton X-100, molecular biology grade (Sigma-Aldrich, cat. no. T8787-250ML)

Sodium Deoxycholate (Sigma-Aldrich, cat. no. 30970-100G)

Ethyl Alcohol, molecular biology grade (Pure; Sigma-Aldrich, cat. no. E7023-500ML)

Tween 20, molecular biology, viscous liquid (Sigma-Aldrich, cat. no. P9416-100ML)

2-Propanol, molecular biology grade (Sigma-Aldrich, cat. no. I9516-500ML)

Phenol:Chloroform:Isoamyl Alcohol 25:24:1 (Saturated with 10mM Tris, pH 8.0, 1mM EDTA; Sigma-Aldrich, cat. no. P3803-400ML)

SDS (10% (wt/vol); Ambion, cat. no. AM9822)

NaCl Solution (5.0 M; Ambion, cat. no. AM9759)

EDTA (0.5 M, pH 8.0; Ambion, cat. no. AM9261)

SSC (20X; Ambion, cat. no. AM9763)

Tris (1 M, pH 8.0; Ambion, cat. no. AM9856)

TE Buffer (pH 8.0; Ambion, cat. no. AM9849)

Sodium Acetate (3M, pH 5.5; Ambion, cat. no. AM9740)

Nuclease-Free water (not DEPC-Treated; Ambion, cat. no. AM9937)

HEPES Buffer (1 M, pH 7.3; Thermo Fisher Scientific, cat. no. BP299-1)

I-Block Protein-Based Blocking Reagent (Thermo Fisher Scientific, cat. no. T2015)

Dynabeads M-280 Streptavidin (Thermo Fisher Scientific, cat. no.11205D)

GlycoBlue Coprecipitant (Thermo Fisher Scientific, cat. no. AM9516)

Proteinase K (Fungal; Thermo Fisher Scientific, cat. no. 25530015)

Qubit dsDNA HS kit (Thermo Fisher Scientific, cat. no. Q32851)

Salmon Sperm DNA Solution (Thermo Fisher Scientific, cat. no. 15632011)

NEBuffer 2 (NEB, cat. no. B7002S)

HaeIII (NEB, cat. no. R0108L)

dATP Solution (NEB, cat. no. N0440S)

dNTP Solution Mix (NEB, cat. no. N0447L)

Klenow Fragment (3′->5′ exo-) (NEB, cat. no. M0212L)

T4 DNA Ligase Reaction Buffer (NEB, cat. no. B0202S)

T4 Polynucleotide Kinase (NEB, cat. no. M0201L)

T4 DNA Polymerase (NEB, cat. no. M0203L)

Quick Ligation Kit (NEB, cat. no. M2200L)

T4 DNA Ligase (NEB, cat. no. M0202L)

Lambda Exonuclease (NEB, cat. no. M0262L)

Exonuclease I (*E. coli*; NEB, cat. no. M0293L)

DNA Polymerase I, Large (Klenow) Fragment (NEB, cat. no. M0210L)

Q5 Hot Start High-Fidelity DNA Polymerase (NEB, cat. no. M0493L)

MaXtract High Density (Qiagen, cat. no. 129056)

QIAquick PCR Purification Kit (Qiagen, cat. no. 28106)

Buffer EB (250 ml; Qiagen, cat. no. 19086)

D-PBS, without calcium and magnesium (1X; Wisent, cat. no. 311-425-CL)

Complete EDTA-free Protease Inhibitor Cocktail (Roche, cat. no. 04693132001)

Agilent DNA High-Sensitivity Kit (Agilent Technologies, cat. no. 5067-4626)

AMPure XP (Beckman, cat. no. A63881)

DNA LoBind Tubes (Eppendorf, cat. no. 022431021)

Covaris Microtube (Covaris, cat. no. 520045)

Reagent Solutions

0.1% (wt/vol) SDS BL-Hi-C Lysis Buffer

50 mM HEPES-KOH, pH 7.5; 150 mM NaCl; 1 mM EDTA; 1% Triton X-100; 0.1% Sodium Deoxycholate; 0.1% SDS

1% (wt/vol) SDS BL-Hi-C Lysis Buffer

50 mM HEPES-KOH, pH 7.5; 150 mM NaCl; 1 mM EDTA; 1% Triton X-100; 0.1% Sodium Deoxycholate; 1% SDS

1× TNE Buffer

10 mM Tris-HCl pH 7.5; 0.1 mM EDTA; 50 mM NaCl

2× Binding and Washing Buffer (2× B&W)

10 mM Tris-HCl pH 7.5; 1 mM EDTA; 2 M NaCl

1× Binding and Washing Buffer (1× B&W)

5 mM Tris-HCl pH 7.5; 500 μM EDTA; 1 M NaCl

1× Tween Wash Buffer (1× TWB)

5 mM Tris-HCl pH=7.5; 500 μM EDTA; 1 mM NaCl, 0.05% Tween 20

1× I-Block Buffer

2% (w/v) I-Block Protein-Based Blocking Reagent; 0.5% SDS

Primers

One-step Bridge Linker:

Bridge Linker S1-F: /5P/GCTGAGGGA/iBiodT/C

Bridge Linker S1-R: CCTCAGCT

Two-step Bridge Linker:

Bridge Linker S2-F: /5P/CGCGATATC/iBIOdT/TATCTGACT

Bridge Linker S2-R: /5P/GTCAGATAAGATATCGCGT

Y-Adaptor:

Y-Adaptor-F: /5P/GATCGGAAGAGCACACGTCTGAACTCCAGTCAC

Y-Adaptor-R: TACACTCTTTCCCTACACGACGCTCTTCCGATCT

PCR primer:

Universal primer 1.0:

AATGATACGGCGACCACCGAGATCTACACTCTTTCCCTACACGAC

Index primer 1.0:

CAAGCAGAAGACGGCATACGAGAT/CGTGATGT/GACTGGAGTTCAGACGTGT (/index/)

NTR-F: AATGATACGGCGACCACCGA

NTR-R: CAAGCAGAAGACGGCATACGA

Equipment

Agilent 2100 Bioanalyzer (Agilent Technologies, cat. no. G2940CA)

Centrifuge (Eppendorf 5810R, cat. no. 22628180)

Centrifuge (Eppendorf 5424R, cat. no. 022620498)

Eppendorf Thermomixer comfort (Eppendorf, cat. no. 5355000038)

DynaMag-2 Magnet (Life Technologies, cat. no. 12321D)

Qubit 2.0 Fluorometer (Invitrogen, cat. no. Q32866)

Covaris Sonicator (Covaris, S220)

ChIA-PET2 Software for data processing

Procedure

1. Adaptor and Bridge Linker Preparation:

a) For Bridge Linker S1, add 1× TNE Buffer to dissolve Bridge Linker S1-F and Bridge Linker S1-R to a concentration of 100 μM, and prepare the mixture with the molecular ratio of 1:1 (*e.g.*, 10 μl of Bridge Linker S1-F and 10 μl of Bridge Linker S1-R) or the optimized ratio. For Bridge Linker S2, add 1× TNE Buffer to dissolve Bridge Linker S2-F and Bridge Linker S2-R to a concentration of 100 μM, and prepare the mixture with the molecular ratio of 1:1 or the optimized ratio. For the Y-Adaptor, add 1× TNE Buffer to dissolve Y-Adaptor-F and Y-Adaptor-R to a concentration of 100 μM, and prepare the mixture with the molecular ratio of 1:1 or the optimized ratio.

b) Run the annealing program for Bridge Linker S1, Bridge Linker S2 and Y-Adaptor on the PCR machine as follows: 95°C for 2 minutes; ramp from 95°C to 75°C (rate:

0.1°C/second) and hold at 75°C for 2 minutes; ramp from 75°C to 65°C (rate: 0.1°C/second) and hold at 65°C for 2 minutes; ramp from 65°C to 50°C (rate: 0.1°C/second) and hold at 50°C for 2 minutes; ramp from 50°C to 37°C (rate: 0.1°C/second) and hold at 37°C for 2 minutes; ramp from 37°C to 20°C (rate: 0.1°C/second) and hold at 20°C for 2 minutes; ramp from 20°C to 4°C (rate: 0.1°C/second) and hold at 4°C indefinitely until collection.

c) Dilute the annealed Bridge Linker S1 and annealed Bridge Linker S2 separately to 200 ng/μl with 1× TNE Buffer. Dilute the annealed Y-Adaptor to 20 μM with 1× TNE Buffer. Then, divide the adaptor and bridge linkers into aliquots for storage at -20°C.

2. Cell Crosslinking

a) Prepare previously grown cells (preferably 5×10^4 to 5×10^6) in fresh full medium with 5×10^5 cells per milliliter at room temperature in a 1.6 ml or 10 ml centrifuge tube (1 ml of medium is recommended).

b) Add 278 μl of 37% formaldehyde solution to obtain a final concentration of 1% v/v in 1 ml of medium. Gently shake for exactly 10 minutes at room temperature to perform fixation.

c) Add 894 μl of 2.5 M glycine to obtain a final concentration of 0.2 M to quench the fixation. Gently shake for 10 minutes at room temperature and 5 minutes on ice.

d) Centrifuge the tube at 300 × *g* for 5 minutes at 4°C (the force is dependent on the cell size), and gently wash the pellet twice with 1 ml of ice-cold 1× PBS. The cell pellet can be stored at -80°C for up to one year.

3. Cell Lysis

a) Resuspend the crosslinked cell pellet in 1 ml of 0.1% (wt/vol) SDS BL-Hi-C Lysis Buffer containing protease inhibitors, and gently shake for 15 minutes at 4°C.

b) Centrifuge the tube at 800 × *g* for 5 minutes at 4°C (the force is dependent on the cell size), and remove the supernatant.

c) Repeat steps a-b once.

d) Resuspend the pellet in 1 ml of 1% (wt/vol) SDS BL-Hi-C Lysis Buffer containing protease inhibitors, and gently shake for 15 minutes at 4°C (the time is dependent on the cell type).

e) Centrifuge the tube at 3000 × *g* for 5 minutes at 4°C (the force is dependent on the cell size), and remove the supernatant.

f) Wash the pellet once with 1 ml of 0.1% (wt/vol) SDS BL-Hi-C Lysis Buffer containing protease inhibitors.

g) Centrifuge the tube at 3000 × *g* for 5 minutes at 4°C. The cell pellet can be stored at -80°C for up to one year.

4. Restriction Digestion

a) Resuspend the cell pellet in 50 µl of 0.5% SDS and shake at 900 r.p.m. for exactly 10 minutes at 62°C on the thermomixer.

b) Add 145 µl of ddH_2O and 25 µl of 10% (v/v) Triton X-100 to a final concentration of 1% (v/v) to quench the SDS. Gently shake for 15 minutes at 37°C.

c) Add 25 µl of 10× NEBuffer 2 and 100 U of HaeIII. Shake at 900 r.p.m. for 2-16 hours (overnight recommended) at 37°C on the thermomixer.

d) Add 2.5 µl of 10 mM dATP solution and 2.5 µl of Klenow Fragment (3′->5′ exo-) for A-tailing. Shake at 900 r.p.m. for 40 minutes at 37°C on the thermomixer.

For restriction enzymes that generate overhang, perform steps e-h instead of steps c-d.

e) Add 25 µl of 10× NEBuffer 2 and 100 U of MboI (or HindIII). Shake at 900 r.p.m. for 2-16 hours (overnight recommended) at 37°C on the thermomixer. Then, incubate at 62°C for 20 minutes.

f) Add 36 µl of ddH$_2$O, 1.5 µl of 10 mM dNTP and 8 µl of Large (Klenow) Fragment. Shake at 900 r.p.m. for 45 minutes at 37°C on the thermomixer.

g) Centrifuge the tube at 3500 × *g* for 5 minutes at room temperature, and remove the supernatant.

h) Resuspend the pellet with 250 µl of ddH2O, 25 µl of NEBuffer 2, 2.5 µl of 10 mM dATP solution and 2.5 µl of Klenow Fragment (3′->5′ exo-) for A-tailing. Shake at 900 r.p.m. for 40 minutes at 37°C on the thermomixer.

5. Proximity Ligation

For two-step proximity ligation, perform steps a-c.

a) Add 750 µl of ddH$_2$O, 120 µl of 10× T4 DNA ligase buffer, 100 µl of 10% (v/v) Triton X-100, 12 µl of 100× BSA, 5 µl of T4 DNA ligase, and 4 µl of 200 ng/µl Bridge Linker S2 for two-step proximity ligation. Gently shake for exactly 4 hours at 16°C on a rotating wheel.

b) Centrifuge the tube at 3500 × *g* for 5 minutes at 4°C, and remove the supernatant.

c) Resuspend the pellet with 309 µl of ddH$_2$O, 35 µl of Lambda Exonuclease Buffer, 3 µl of Lambda Exonuclease, and 3 µl of Exonuclease I to remove unligated Bridge Linker. Shake at 900 r.p.m. for 1 hour at 37°C on the thermomixer.

For one-step proximity ligation, perform steps d-j.

d) Add 745 µl of ddH$_2$O, 120 µl of 10× T4 DNA ligase buffer, 100 µl of 10% (v/v) Triton X-100, 12 µl of 100× BSA, 5 µl of T4 DNA ligase, and 9 µl of 200 ng/µl Bridge Linker S1 for linker ligation. Gently shake for exactly 4 hours at 16°C on a rotating wheel.

e) Centrifuge the tube at 3500 × *g* for 5 minutes at 4°C, and remove the supernatant.

f) Resuspend the pellet with 170 µl of ddH_2O, 20 µl of 10× T4 DNA ligase buffer, and 10 µl of T4 Polynucleotide Kinase for linker phosphorylation. Shake at 900 r.p.m. for 1 hour at 37°C on the thermomixer.

g) Centrifuge the tube at 3500 × g for 5 minutes at 4°C, and remove the supernatant.

h) Resuspend the pellet with 755 µl of ddH_2O, 120 µl of 10× T4 DNA ligase buffer, 100 µl of 10% (v/v) Triton X-100, 12 µl of 100× BSA, and 5 µl of T4 DNA ligase for one-step proximity ligation. Gently shake for exactly 4 hours at 16°C on a rotating wheel.

i) Centrifuge the tube at 3500 × *g* for 5 minutes at 4°C, and remove the supernatant.

j) Resuspend the pellet with 309 µl of ddH_2O, 35 µl of Lambda Exonuclease Buffer, 3 µl of Lambda Exonuclease, and 3 µl of Exonuclease I to remove unligated Bridge Linker. Shake at 900 r.p.m. for 1 hour at 37°C on the thermomixer.

6. DNA Purification

a) Add 45 µl of 10% SDS and 55 µl of 20 mg/ml Proteinase K for crosslinking reversal. Incubate at 55°C for at least 2 hours (overnight recommended). Then, add 65 µl of 5 M NaCl and incubate at 68°C for 2 hours.

b) Add 500 µl of phenol:chloroform:isoamyl alcohol 25:24:1 and mix well. Then, transfer mixture to a 2 ml MaXtract High Density tube to separate the aqueous phases.

c) Add 1 µl of GlycoBlue, 50 µl of 3 M sodium acetate, pH 5.2, and 550 µl of isopropanol. Incubate the mixture at -80°C for 1 hour.

d) Centrifuge the mixture at maximum speed for 30 minutes at 4°C, and remove the supernatant. Then, wash the pellet twice with ice-cold 75% ethyl alcohol.

e) Resuspend the dried pellet with 130 µl of Buffer EB.

7. Sonication

a) Transfer the DNA solution into a Covaris tube.

b) Set Covaris parameters for the DNA size of 400 bp, and sonicate.

8. Biotin Pull-Down

a) Transfer 30 μl of M280 Streptavidin Dynabeads suspension to a 1.6-ml tube for pre-preparation. Wash the Dynabeads twice with 100 μl of 2× B&W buffer.

b) Resuspend the Dynabeads with 100 μl of 1× I-Block buffer, and mix for 45 minutes at room temperature on a rotating wheel.

c) Wash the Dynabeads twice with 100 μl of 1× B&W buffer.

d) Resuspend the Dynabeads with 100 μl of 1× B&W buffer containing 1 μg of Salmon Sperm DNA solution, and mix for 30 minutes at room temperature on a rotating wheel.

e) Wash the Dynabeads twice with 100 μl of 1× B&W buffer.

f) Transfer the sonicated DNA solution with 130 μl of 2× B&W buffer to the pre-prepared Dynabeads, and mix for 45 minutes at room temperature on a rotating wheel.

g) Wash the DNAs-on-Dynabeads five times with 500 μl of 2× SSC/0.5% (w/v) SDS.

h) Wash the DNAs-on-Dynabeads twice with 500 μl of 1× B&W buffer.

f) Wash the DNAs-on-Dynabeads once with 100 μl of Buffer EB.

9. Library Construction

a) Add 75 μl of ddH_2O, 10 μl of 10×T4 DNA Ligase Buffer, 5 μl of 10 mM dNTP, 5 μl of T4 Polynucleotide Kinase, 4 μl of T4 DNA Polymerase and 1 μl of Large

(Klenow) Fragment to the DNAs-on-Dynabeads for end repair. Shake at 900 r.p.m. for 30 minutes at 37°C on the thermomixer.

b) Wash the DNAs-on-Dynabeads twice with 500 µl of 1× TWB for 2 minutes at 55°C.

c) Add 80 µl of ddH_2O, 10 µl of 10× NEBuffer 2, 5 µl of 10 mM dATP and 5 µl of Klenow Fragment (3′->5′ exo-) to the DNAs-on-Dynabeads for A-tailing. Shake at 900 r.p.m. for 30 minutes at 37°C on the thermomixer.

d) Wash the DNAs-on-Dynabeads twice with 500 µl of 1× TWB for 2 minutes at 55°C.

e) Wash the DNAs-on-Dynabeads once with 50 µl of 1× Quick Ligase Buffer.

f) Add 6.6 µl of ddH_2O, 10 µl of 2× Quick Ligase Buffer, 2 µl of Quick Ligase and 0.4 µl of 20 µM Y-Adaptor for sequencing adaptor ligation. Incubate for 15 minutes at room temperature.

g) Wash the DNAs-on-Dynabeads twice with 500 µl of 1× TWB for 2 minutes at 55°C.

h) Wash the DNAs-on-Dynabeads once with 100 µl of Buffer EB.

i) Resuspend the DNAs-on-Dynabeads with 60 µl of Buffer EB, and divide into 20 µl aliquots for storage at -20°C.

10. PCR Amplification

a) Prepare PCR mix as follows: 20 µl of DNAs-on-Dynabeads, 8.5 µl of ddH_2O, 10 µl of Q5 Reaction Buffer, 1 µl of 10 mM dNTP, 0.5 µl of Q5 Hot Start High-Fidelity DNA Polymerase, 2.5 µl of Universal Primer 1.0, 2.5 µl of Index Primer 1.0, 2.5 µl of NTR-F, and 2.5 µl of NTR-R.

b) Run the PCR program according to the instructions for Q5 DNA Polymerase on the PCR machine with 9-12 amplification cycles.

c) Transfer the PCR products without Dynabeads to a 1.6-ml tube. Then, add 30 µl of AMPure Beads to achieve a 0.6:1 volumetric ratio of AMPure Beads + sample. Mix well, and incubate for 5 minutes at room temperature.

d) Place the tube on the magnetic rack, and transfer the supernatant to a 1.6-ml tube. Then, add 7.5 µl of AMPure Beads to achieve a 0.75:1 volumetric ratio of AMPure Beads + sample. Mix well, and incubate for 5 minutes at room temperature.

e) Place the tube on the magnetic rack, and discard the supernatant.

f) Keep the tube on the magnetic rack, wash the AMPure Beads twice with 200 µl of 80% ethyl alcohol.

g) Dry the AMPure Beads, and elute the DNA with 20 µl of ddH_2O for storage at -20°C.

11. Sequencing

a) Measure the BL-Hi-C library with a Qubit Fluorometer and Agilent 2100 Bioanalyzer.

b) Sequence the BL-Hi-C library with the Illumina Sequencer HiSeq X Ten (PE 2× 150 bp reads).

12. Data Processing

a) Run ChIA-PET2 software with the parameters "-m 1 -k 2 -e 1 -A ACGCGATATCTTATC -B AGTCAGATAAGATAT" for two-step proximity ligation.

b) Run ChIA-PET2 software with the parameters "-m 2 -k 2 -e 1 -A AGCTGAGGGATCCCT -B AGCTGAGGGATCCCT" for one-step proximity ligation.

Supplementary References

1. Rao, S.S., et al., *A 3D map of the human genome at kilobase resolution reveals principles of chromatin looping.* Cell, 2014. **159**(7): p. 1665-80.
2. Tang, Z., et al., *CTCF-Mediated Human 3D Genome Architecture Reveals Chromatin Topology for Transcription.* Cell, 2015. **163**(7): p. 1611-27.
3. Heinz, S., et al., *Simple combinations of lineage-determining transcription factors prime cis-regulatory elements required for macrophage and B cell identities.* Mol Cell, 2010. **38**(4): p. 576-89.
4. Mumbach, M.R., et al., *HiChIP: efficient and sensitive analysis of protein-directed genome architecture.* Nat Methods, 2016. **13**(11): p. 919-922.
5. Ernst, J. and M. Kellis, *ChromHMM: automating chromatin-state discovery and characterization.* Nat Methods, 2012. **9**(3): p. 215-6.
6. Ashoor, H., et al., *DENdb: database of integrated human enhancers.* Database (Oxford), 2015. **2015**.
7. Consortium, E.P., *The ENCODE (ENCyclopedia Of DNA Elements) Project.* Science, 2004. **306**(5696): p. 636-40.
8. Mullard, A., *The Roadmap Epigenomics Project opens new drug development avenues.* Nat Rev Drug Discov, 2015. **14**(4): p. 223-5.
9. Li, G., et al., *ChIA-PET2: a versatile and flexible pipeline for ChIA-PET data analysis.* Nucleic Acids Res, 2017. **45**(1): p. e4.
10. Li, X., et al., *Long-read ChIA-PET for base-pair-resolution mapping of haplotype-specific chromatin interactions.* Nat Protoc, 2017. **12**(5): p. 899-915.
11. Ramani, V., et al., *Mapping 3D genome architecture through in situ DNase Hi-C.* Nat Protoc, 2016. **11**(11): p. 2104-21.
